# Electrical stimulation for cartilage tissue engineering - A critical review from an engineer's perspective

**DOI:** 10.1016/j.heliyon.2024.e38112

**Published:** 2024-09-23

**Authors:** Julius Zimmermann, Abdul Razzaq Farooqi, Ursula van Rienen

**Affiliations:** a Institute of General Electrical Engineering, University of Rostock, 18051 Rostock, Germany; b Department of Electronic Engineering, Faculty of Engineering, The Islamia University of Bahawalpur, 63100 Bahawalpur, Pakistan; c Department of Ageing of Individuals and Society, Interdisciplinary Faculty, University of Rostock, 18051 Rostock, Germany; d Department Life, Light & Matter, University of Rostock, 18051 Rostock, Germany

**Keywords:** Articular cartilage, Cartilage tissue engineering, Electrical stimulation, Stimulation devices, Numerical simulations, Biophysical mechanisms

## Abstract

Cartilage has a limited intrinsic healing capacity. Hence, cartilage degradation and lesions pose a huge clinical challenge, particularly in an ageing society. Osteoarthritis impacts a significant number of the population and requires the development of repair and tissue engineering methods for hyaline articular cartilage. In this context, electrical stimulation has been investigated for more than 50 years already. Yet, no well-established clinical therapy to treat osteoarthritis by means of electrical stimulation exists. We argue that one reason is the lack of replicability of electrical stimulation devices from a technical perspective together with lacking hypotheses of the biophysical mechanism. Hence, first, the electrical stimulation studies reported in the context of cartilage tissue engineering with a special focus on technical details are summarized. Then, an experimental and numerical approach is discussed to make the electrical stimulation experiments replicable. Finally, biophysical hypotheses have been reviewed on the interaction of electric fields and cells that are relevant for cartilage tissue engineering. With that, the aim is to inspire future research to enable clinical electrical stimulation therapies to fight osteoarthritis.

## 1 Introduction

Cartilage is found in the human body as hyaline, elastic, and fibrocartilage, each of which performs different functions [1]. Among the three cartilaginous tissues, hyaline cartilage is of great clinical importance because it protects the bones during articulation and acts as a shock absorber in the knee joint [2], [3]. If it is damaged or degraded, for example, in osteoarthritis, articulation becomes painful, and eventually, the subchondral bone will wear off. The majority of the population of ageing societies can be expected to suffer from osteoarthritis. Moreover, joint degeneration is common among athletes through damage to the articular cartilage due to trauma, repetitive impact and loading. However, current surgical cartilage repair techniques are limited and are mostly considered for defects of less than  [4]. Eventually, severe cases of osteoarthritis require a total joint replacement. An increasing number of joint replacements already evidences this aspect [5]. Tissue engineering approaches could pave the way towards successful interventions at an early stage of the disease. Furthermore, it shall become possible to treat large area defects surgically [4].

At first glance, the engineering of cartilage tissue might appear straightforward: only one type of cell is present while there is no vascular network in cartilage. Moreover, the shape of the tissue to be replaced is rather simplistic (usually disk-like geometries). However, an ‘ideal’ approach for cartilage tissue engineering is still elusive [6], [7].

Electrical stimulation can be applied in two possible scenarios: monolayer or two-dimensional (2D) and three-dimensional (3D). Both of these scenarios are important. The monolayer chondrocyte loses its morphophysiological characteristics, so 3D environments are preferred. However, 3D scenarios add complexity to cell culture. For example, high-resolution screening and monitoring is an important challenge in the case of 3D cell cultures [8].

**Figure 1:**
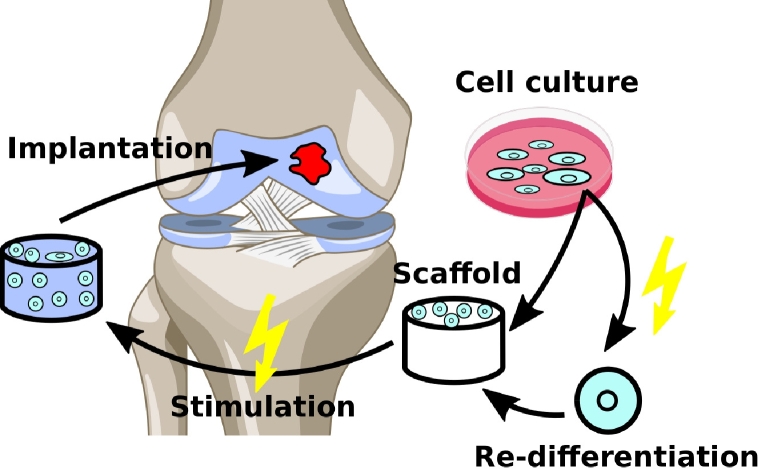
Summary of the cartilage regeneration using the tissue engineering approach. Cells are expanded and seeded on a scaffold before implantation at the defect site. During cell cultivation in a monolayer, the cell morphology can change due to dedifferentiation. To ensure articular cartilage formation, the chondrogenic phenotype of the cells should be preserved. Electrical stimulation can benefit re-differentiation [15] as well as tissue formation in 3D cell culture. This figure has been derived from an image by the Database Center for Life Science (DBCLS), used under Creative Commons Attribution 4.0 International.

Approaches to engineer cartilage have focused on various scaffold materials, cells and biological or biophysical stimulation signals [9]. In recent years, matrix-assisted autologous chondrocyte implantation (MACI) as a tissue engineering approach has successfully been clinically translated [10]. Initial clinical studies have shown that MACI can be applied on defects larger than  [11]. Current research directions investigate the use of cells other than autologous chondrocytes (e.g., mesenchymal stem cells (MSCs)) and biophysical or chemical stimulation of cell-seeded scaffolds to improve MACI (Fig. 1) [12], [13]. Moreover, scaffold-free approaches are investigated but are prone to the dedifferentiation of chondrocytes, which is a drawback of 2D cell culture (i.e., cells grown in a monolayer) [9], [14], [15]. Upon dedifferentiation, collagen type I or type X are produced instead of collagen type II as the chondrocytes lose their chondrogenic phenotype.

3D cell culture approaches usually make use of scaffolds. Hydrogels as scaffold material resemble the native extracellular matrix [16] and have thus become a popular choice. They can be seeded with cells, but cell-free approaches based on scaffolds doped with growth factors have been explored [17]. From a bioengineering point of view, hydrogels can enable patient-specific cartilage repair implants because they can be 3D-printed [18], [19].

A challenge for the engineering of hyaline cartilage is that the produced tissue does often not have the desired mechanical properties but resembles fibrocartilage [6], [9], [20]. Thus, it is paramount to develop an approach that produces engineered tissue resembling hyaline cartilage. Biophysical stimulation (e.g., mechanical and electromagnetic stimulation) has been explored to foster cell proliferation or differentiation before implantation [21]. The reasoning for mechanical stimulation is to mimic the cyclic loading during walking. Electrical stimulation is explored because it has been found that mechanical signals induce currents in the vicinity of chondrocytes [22], [23]. However, it is an open question which stimulation parameters benefit cartilage tissue generation.

Electromagnetic stimulation of cartilage can be divided into two approaches: pulsed electromagnetic field (PEMF) stimulation and electrical stimulation. In PEMF, an externally applied magnetic field induces an electric current in the specimen [24]. In contrast, electrical stimulation involves methods that use only electrical signals (voltage, current) to expose the sample to an electric field. While PEMF has been reported to reduce an inflammatory response [21], [25], it cannot provide localised stimulation and may lead, for example, to the growth of adjacent bone [26]. In contrast, electrical stimulation can be applied to selected local regions. In this review, we will, therefore, exclusively focus on electrical stimulation.

Electrical stimulation has also been considered for regenerative medicine [27], [28] in fields such as cardiac tissue engineering [29], wound healing [30], [31], [32], bone regeneration [33] or neural stimulation [34]. Various in vitro electrical stimulation approaches have been reviewed [21], [27], [28], [35], [36], [37], [38]. Still, the reasons for the therapeutic effect of most electrical stimulation approaches remain elusive. To pave the way for future developments in the field of cartilage tissue engineering and repair, we aim to provide an overview of the potential fundamental biophysical aspects of the electrical stimulation of cartilage. A challenge is that, to date, the focus of the research is usually on the biological effects, but a discussion of the technical parameters is lacking. However, standardised, replicable electrical stimulation approaches are required to cope with the plethora of stimulation parameters and rule out external influences. The open challenges lie in particular in the interdisciplinary character of the field: Researchers with a background in biology, medicine, physics, mechanical or electrical engineering meet.

The aim of this paper is to explain stimulation approaches for cartilage tissue engineering using electrical stimulation from an engineer's perspective. We will summarise the status quo such that readers without a background in electrical engineering can follow. Based on the literature review, a relation will be formulated between stimulation experiments and numerical simulations, which permit a precise and reliable estimation of the stimulating electric field. Finally, we conclude with an overview of biophysical theories that can be used to develop hypotheses for the stimulation effect in cartilage tissue engineering. This review is largely based on the work done in the scope of the doctoral theses of two of the authors [39], [40].

## 2 The use of electric fields in cartilage engineering and repair

Electrical stimulation for cartilage tissue engineering has a long tradition dating back to the 1970s [41]. This section provides an overview of the different in vitro and in vivo studies and their reported biological outcome. The aim is to introduce the specific objectives of cartilage engineering and repair and how they are assessed in vitro and in vivo. The technical aspects will be mentioned only briefly and will be discussed in more detail in the next section.

### 2.1 In vitro stimulation

Many in vitro studies have focused on the influence of capacitive coupling stimulation. Brighton's group is considered a pioneer in this research area and developed a stimulation protocol [42], [43], which has been reported to increase extracellular matrix synthesis [44], [45]. Later, the same stimulation protocol was modified by other research groups who noted that a similar protocol could enhance redifferentiation [15] or improve differentiation of stem cells into chondrocytes [46]. Redifferentiation is an essential aspect because cartilage cells grown in a monolayer tend to dedifferentiate (i.e., convert to fibroblast) [14], [15]. Growing chondrocytes on scaffolds (3D cell culture) also supports redifferentiation [14]. Thus, the combination of cell-seeded scaffolds and electrical stimulation is the subject of current research [47]. Kwon et al. reported the differentiation of MSCs into hyaline chondrocytes in culture upon electrical stimulation in the absence of growth factors [48]. This is an important aspect, as growth factors are commonly used to support cartilaginous differentiation. If electrical stimulation works reliably without growth factors compared to the control group, it is a very clear sign of its efficacy. When used in combination with growth factors, there should be a clearly observable effect. In a recent review, it was argued that the use of electrical stimulation for osteochondral stem cell regeneration needs more research because of conflicting results [49].

Another goal of electrical stimulation is to induce chondrocyte migration and alignment. For example, direct contact electrical stimulation has been investigated for galvanotactic [50] or dielectric migration [51]. This can be used to improve tissue engineering and repair approaches, which are currently undertaken by modifying the structure and chemistry of hydrogels [52].

### 2.2 In vivo stimulation

Direct contact electric stimulation was applied for cartilage repair in rats [53] and rabbits [54]. Based on the initial investigations of Leppiello et al. [54], gel-covered electrodes were placed on the skin to ensure direct contact with the body for the treatment of knee osteoarthritis [55], [56], [57]. The authors of these studies have concluded that electrical treatment helps improve symptoms and knee function. Conversely, another research group using a similar device has observed no major effect [58]. Differences between the studies were, for example, the stimulation amplitudes. While in one study, a fixed voltage was set [55], another study required patients to adjust the voltage [58]. As there was no reasoning for the chosen stimulation approach given nor an analysis of the impact of the voltage conducted, this example highlights why a detailed analysis of the technical stimulation details is required.

Systematic reviews of clinical studies on the application of electrical stimulation for the treatment of osteoarthritis have revealed a positive effect regarding improved function, but further relevant research is required [59], [60]. Recent work has found that interferential current therapy does not yield a clinical benefit [61]. Similarly, no solid evidence has been reported regarding transcutaneous electrical stimulation's effectiveness for knee osteoarthritis [62]. This unclear situation also exists for electrical stimulation approaches to reach pain relief in osteoarthritis [63].

In short, in vitro experiments target a mechanistic understanding of electrical stimulation for cartilage tissue engineering. The goal is to generate cartilage-like tissue structures, e.g., based on hydrogels, in a fast and reliable manner through electrical stimulation. In vivo experiments focus more on clinical outcomes (e.g., pain relief or function), as the tissue quality cannot be easily assessed in clinical trials. Due to patient-specific tissue properties, it is difficult to estimate the local stimulation field strength. These technical aspects will be addressed in more detail below.

## 3 Comparison and characterisation of stimulation devices

Well-controlled in vitro experiments are needed to comprehensibly investigate the interaction between applied stimulation and the cells [64]. The in vitro experiments should be replicable to ensure transformation into successful clinical translation. In this regard, developing public protocols and standards is crucial [65]. Problems in the documentation and reproducibility of experimental studies using electrical stimulation have been observed in the recently reported studies [66], [67], [68], [69], [70], [71], [72]. In the field of bone regeneration, which is relatively close to cartilage tissue engineering, insufficient device specifications and documentation have been identified as significant reasons why electrical stimulation has not entered standard clinical practice despite more than 60 years of research [69]. To more rapidly achieve the research goal of cartilage regeneration, the current barriers to clinical translation due to a lack of technical specificity should be overcome.

**Figure 2:**
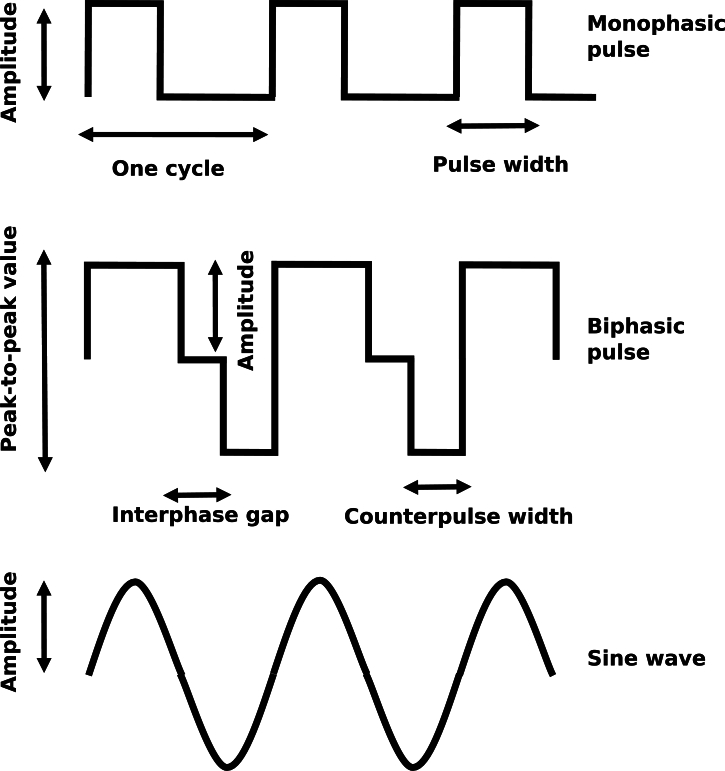
Comparison of the most common waveforms used for electrical stimulation in the context of cartilage tissue engineering. All shown signals are periodic signals. The monophasic and biphasic pulses can be understood as a superposition of sine waves of different frequencies. More details about this representation can be found, for example, in [71]. The exponentially decreasing pulses used in vivo can be understood as monophasic pulses with an exponentially decaying amplitude (see, for example, illustration in [55]).

This section focuses on the electrical characteristics of the typical stimulation techniques in cartilage tissue engineering. In general, two types of stimulation are prevalent: direct contact and capacitive coupling stimulation. Capacitive coupling refers to the presence of a thin, dielectric, non-conducting layer between the electrodes and the sample, while direct contact refers to the absence of the dielectric layer. A voltage is applied to the electrodes, resulting in an electric field or a current density. One can only speak of a voltage if the electric potential is defined with respect to a reference potential (usually the ground, which is set at ). The electric potential is a scalar field. It has no direction and only an amplitude at any point in space. The electric field, on the other hand, is a vectorial quantity, i.e., it has a direction and a magnitude, which is referred to here as the electric field strength. The current density is related to the electric field by the conductivity. The current is defined as the integral of the normal current density passing through a closed surface. Other quantities, such as the charge or charge density, are not commonly reported in the context of cartilage tissue engineering. Different waveforms have been considered for cartilage-tissue engineering (see the comparison of waveforms in Fig. 2). To compare the different studies, the focus was on identifying the employed stimulation protocol, electric field, applied voltage, frequency, and current/current density.

### 3.1 Physiological electric fields and current densities

To give the reader a perspective, we will first briefly outline the physiologically expected electric fields and current densities. A first estimate of the physiologically expected electric field strength can be made based on the works of Bassett and Pawluk [73] and Frank et al. [74]. They have measured streaming potentials in the range of  to  upon compression. The load used by Bassett and Pawluk has followed a rectangular waveform with a pulse width of  (and likewise a not clearly specified frequency in the  range) while Frank et al. have used sinusoidal loads at frequencies between  and . The current densities due to physiological loading have been estimated based on in vitro measurements [74] and theoretical considerations [75] to be about . From this information, the physiological electric field can be estimated. As parallel-plate capacitor-like electrode configurations have been used to measure the streaming potential, the electric field strength can be estimated to be the streaming potential over the sample thickness. Typically, the samples were about  to  thick. This gives an electric field amplitude estimate based on the streaming potential as about  (assuming, for example,  streaming potential and  sample thickness). The electric field strength *E* can also be estimated from the current density *J* and the cartilage conductivity *σ*, which is about  [76], [77]. Then, the field is about(1)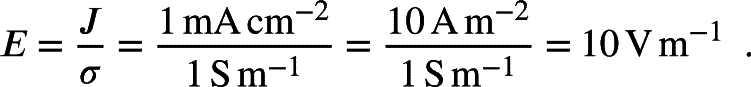 This exemplary calculation based on the literature data shows that the electric field amplitude that comes closest to the presumed physiological electric field amplitude should be in the order of  to . For the sake of comparability, we chose to give the electric fields here per centimetre, which turns the aforementioned range into  to . Note that this range should not be treated as absolute but give an idea of the expected order of magnitude.

### 3.2 Overview of direct contact stimulation approaches

**Figure 3:**
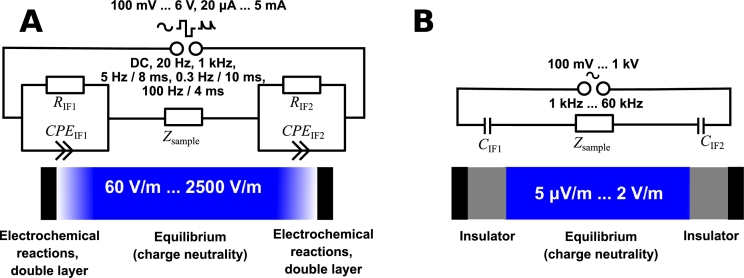
Summary of electrical stimulation techniques and associated equivalent circuit models. We report the range of estimated stimulation field strengths, stimulation amplitudes, frequencies and waveforms for direct contact electric stimulation (panel **A**) and capacitive coupling stimulation (panel **B**). All waveforms are periodic and are either time-harmonic (sine waves) or represented as the Fourier series (rectangular waves, decaying exponentials) [135]. Equivalent circuit models can be used to describe the stimulation devices from an electrochemical point of view: A direct contact stimulation model includes electrochemical processes at the electrode surface (denoted by the resistance *R* and the constant-phase element *CPE* at the two interfaces *IF*1,2, panel **A**). Calibration is required for each electrode and sample and model augmentation may also be required. Alternatively, capacitively coupled electric stimulation can be modelled using existing dielectric and geometric material properties without the need for calibration to predict the sample impedance *Z*_sample_ (panel **B**). Instead of the electrochemical interface impedance, only the capacitance of the dielectric interface layers *C*_IF1,2_ is required. Figure taken from [40].

Direct contact electrical stimulation is the most comprehensible approach as the electrodes are brought in direct contact with the sample and a stimulus is applied (see also the scheme in Fig. 3**A**).

Among the waveforms under consideration, rectangular pulses have been the subject of in vivo studies. For example, a current-controlled  biphasic rectangular pulse of width  having  frequency has been utilized to investigate cartilage repair in adult rats [53]. Similar to deep brain stimulation (DBS) applications, an interphase gap between pulse and counterpulse has been considered. While most of the studies have not measured physical quantities related to the stimulation, Lippiello et al. have reported a maximal current of  at a spike voltage of  through a rabbit's knee when using the  exponentially decreasing signal [54]. Furthermore, an average value of  and peak value of  have been reported when measurement electrodes were placed inside the tissue [54]. Monophasic rectangular pulses of  (in vitro) and  (in vivo) with  pulse width at  have improved matrix metabolism in vitro and in a rat model [78]. In this study, the electrodes were also used to heat the knee. The choice of different parameters for in vitro and in vivo stimulation was not explained. Kwon et al. used biphasic pulses of width  having  frequency with unreported voltage or current to promote chondrogenesis of MSCs [48]. The authors tested the effect of electric field magnitudes in the range  – . A device having “pulsed, asymmetrically biphasic, exponentially decreasing waveform with a frequency of  and pulse width of ” was incorporated to estimate the clinical effectiveness of electrical stimulation [58]. The clinical study participants could set other details (applied voltage or current) that have not been recorded. A similar device having a spike voltage (at the onset of the pulse) of  has been utilized without reporting the electric current value [55]. Please note that the devices tested for clinical use have been placed in direct contact with the skin but not cartilage tissue. We consider this direct-contact stimulation because electrochemical reactions can occur at the electrode-skin interface depending on, for example, the skin wetting.

Another type of stimulation using rectangular signals is nano-second pulsed electrical field (nsPEF) stimulation. High voltage electric signals leading to field strengths in the range of  are applied for a very short duration [79]. This type of electric stimulation triggers biological effects while reducing the cell damage that can be caused by prolonged conventional electric field stimulation [80], [81]. Application of nsPEF to porcine knee articular chondrocytes resulted in enhanced cell proliferation and dedifferentiation as evidenced by relevant gene expressions and signalling pathways [82]. Furthermore, it has been reported that sequential application of nsPEF followed by treatment with ghrelin peptide increases the chondrogenesis of rat MSCs in vitro and eventually enhances regeneration of osteochondral tissue [83]. In an early work, Rodan et al. have used similarly strong fields with much slower pulses (i.e., longer exposure) to open the membrane [84].

Aside from rectangular pulses, sinusoidal waves of low frequency have been considered for direct contact stimulation. e.g.,  for the in vitro stimulation of chondrocytes [85] while  are common in the related field of bone regeneration [86], [87]. The voltage amplitude of a few hundred  to  have been incorporated in these experiments, while the effective currents have yet to be reported. The electric field estimation in the cell culture medium was performed using local voltage measurements [87]. In the research group of Grodzinsky et al., various electrical stimulation experiments using sine wave signals have been conducted in the context of cartilage tissue engineering. Initially, in vitro stimulation of chondrocytes at low frequencies ranging from  to  has led to no response [88]. Later, increased protein synthesis in articular cartilage explants at higher frequencies ( and ) has been shown [89], [90]. Sine wave stimulation at frequencies between  and  has shown upregulated cell proliferation together with the synthesis of extracellular matrix [91]. The effect was present at all frequencies, with the best result at . Preliminary experiments have shown that direct current (DC) stimulation leads to significantly increased temperatures and exposure to electrochemical reaction products. In all works of this group, the imposed current density has been reported. However, this quantity is not provided by a power source but it is a derived quantity that depends on the electrode surface area. The actual value provided by the power source in the experiment has not been reported. In addition to electrical stimulation, Grodzinsky's group has experimentally and theoretically studied electrokinetic transduction phenomena in, for example, bovine knee cartilage samples [74], [75]. These studies were used at the beginning of Sec. 3 to estimate the physiological electric field strengths. Similarly, optical techniques have been used to study electrokinetic effects [92], [93] and cartilage degeneration has been detected using electromechanical surface spectroscopy [94]. Other groups have electrified the substrate on which the cells are grown [95]. Furthermore, frequencies as high as  have been investigated [96]. However, such studies have not yet been conducted by multiple groups.

Experimental approaches in tissue engineering also exist that use DC signals [36]. The direct current is mainly the result of faradaic electrochemical reactions [97]. Agar-salt bridges are usually employed in DC experiments to protect the cell culture from chemical byproducts produced due to the electrochemical reactions occurring at the electrodes [36]. Direct current signals are also incorporated to explore the galvanotaxis or electrotaxis effect. For example, an electric current of around  has been reported to start the chondrocyte cathodal migration, and the authors have also compared the expected and measured values of the chamber's resistance [50]. Furthermore, they have estimated the magnitude of the electric field strength (up to ) by measuring the voltage difference between two electrodes. Earlier, Nogami et al. have found pronounced formation of cartilage at the cathode [98]. Baker et al. have reported that the limited regeneration capability of articular cartilage can be increased by using DC stimulation [99] or DC voltages induced by galvanic potentials [41]. For the latter, two wires made of different materials were used to impose the voltage but no active stimulator was used, i.e., the stimulation was passive. In contrast, DC stimulation of chondrocytes in 3D agarose culture has not led to a significant response [100].

All the aforementioned in vitro and in vivo studies of direct contact electrical stimulation are summarised in Table 1.

**Table 1:** Electrical stimulation studies of articular cartilage using direct coupling. Please note that consistent units were used (either  or ) to make the studies comparable.

Study	Year	Stimulation protocol	Electric Field Strength	Voltage	Frequency	Current / Current density	Waveform	Tissue type / Cell line
**in vivo studies**								

Baker et al. [99]	1974	1 – 9 weeks	NR	15 –	NA		DC	Rabbit knee
Baker et al. [41]	1974	2 – 3 weeks	NR	40 –	NA		DC, passive	Rabbit knee
Lippiello et al. [54]	1990	, 5 days/week for 2 – 8 weeks	20 –				Exponentially decaying spike, PW	Rabbit knee
Farr et al. [119]	2006	Around	NR	up to		Monophasic, spiked	NR	Human knee
Garland et al. [56]	2007	h/day for 12 weeks	NR	0 –		Monophasic, spiked	NR	Human knee
Fary et al. [58]	2011	26 weeks	NR	NR		NR	Biphasic, spiked, PW	Human knee
Hungerford et al. [55]	2013	1 – 12 months	NR			NR	Monophasic, spiked	Human knee
Hiraoka et al. [78]	2013		NR			NR	Monophasic pulse, PW	Rat knee
Zuzzi et al. [53]	2013	for 7, 21, or 35 days	NR	NR			Biphasic pulse, PW , IG	Rat non-articular cartilage

**in vitro studies**								

Rodan et al. [84]	1978	2, 4, or		0 and		NR	Rectangular pulse, PW	Chick chondrocytes
Nogami et al. [98]	1982	1, 2, or 3 weeks	NR	NR	NA		NR	Rat MSCs
Gray et al. [88]	1986		NR	NR	0.1 –	50 – (≈ – )	Sine wave	Calf epiphyseal plate cartilage
MacGinitie et al. [89], [90]	1987, 1994		NR	NR	1 –	10 –	Sine wave	Calf articular chondrocytes in agarose gel
Chao et al. [50]	2000	More than an hour	170, 800, 2000, 4000, or	NR	NA	, 0.003, 0.012, 0.03, 0.07, or	NR	Bovine articular chondrocytes
Szasz et al. [91]	2003		NR	NR	–		Sine wave	Calf chondrocytes in agarose gel
Akanji et al. [100]	2008	6 or	NR	NR	NA	2, 4, or	NR	Steer chondrocytes in agarose gel
Khang et al. [95]	2008	3 or	NR	NR			NR	Human chondrocytes
Hiraoka et al. [78]	2013		NR			NR	Monophasic pulse, PW	Rabbit chondrocytes
Zhang et al. [82]	2014	5 pulses of	10 or	NR		NR	Pulse signal	Porcine articular chondrocytes
Hernández-Bule et al. [96]	2014	5 min On, Off for	NR	NR			Sine wave	Human ADSCs
Kwon et al. [48]	2016	3 or 7 days	1000, 5000, or	NR		NR	Bipolar pulse,	Mouse MSCs
Hiemer et al. [85]	2018	45 min, 3 times/day for 7 days	200 –			NR	Sine wave	Human articular chondrocytes
Ning et al. [81]	2019	5 pulses	10 or	NR		NR	Monophasic; PW 10, 60, 100, or	Porcine BMMSCs
Chen et al. [79]	2020	5 pulses	10 –	NR		NR	Monophasic; PW 10 –	Porcine BMMSCs
Li et al. [80]	2020	5 pulses	10 or	NR		NR	Monophasic; PW	Human or Porcine MSCs
Li et al. [83]	2022	5 pulses		NR		NR	Monophasic, PW 10, 60, or	Rat MSCs

^⁎^ MSCs, mesenchymal stem cells; ADSCs, adipose-derived stem cells; BMMSCs, bone marrow-derived stromal cells; DC, direct current; NR, not reported; NA, not applicable; PW, pulse width; IG, interphase gap.

### 3.3 Capacitive coupling stimulation

At first, capacitive coupling seems conflicting because no direct current flows between the sample and stimulation electrodes as the current is blocked due to an insulating layer on the stimulation electrodes. Capacitive coupling is widely used for the electrical stimulation of chondrogenic differentiation of adipose-derived stem cells (ADSCs) [101], chondrocytes [15], [102], [103], cartilage [45], osteoblasts [104], and bone [105]. Higher frequencies in the range of  are usually necessary to produce sufficient electric fields in the sample [106], [107], [108], [109]. Thanks to the insulating layer, no electrochemical reactions can occur at the electrode surface. Thus, the capacitive-coupling approach is more biocompatible than direct contact electric stimulation.

For cartilage tissue engineering and regeneration involving capacitive coupling, most of the work has been performed by Brighton's group since the 1980's [42], [44], [45], [103], [110], [111], [112]. Other researchers have also utilized their work [15], [46], [113]. These investigations incorporated capacitive coupling for the electric stimulation of cartilage explants [45], [111], [112] as well as human and animal chondrocytes in the cell culture medium [15], [42], [44], [103], [110], [113]. Based on these studies,  has been established as the preferred frequency for electric signals.

In 2004, a study was reported by Brighton's group using  and  frequencies for capacitive stimulation [44]. The bovine articular chondrocytes were utilized to investigate the effect of electric stimulation on extracellular matrix production for an up-regulation of aggrecan and type II collagen mRNA. At  frequency, a beneficial effect of electric stimulation was obtained to confirm the previously selected frequency. The stimulating electric field strength of  has been reported [42], [44], [45], [103], [111], [112] but no experimental evidence is available for using this value. Unfortunately, the theoretical and experimental investigations of the existing stimulation modalities are hard to find. The voltage of  has been incorporated without explicitly describing if the reported value is root-mean-square or peak-to-peak voltage [43]. In an early edition of this stimulation device, a thicker layer of insulation on electrodes was incorporated, necessitating up to  peak-to-peak voltage [110].

In recent years, other frequencies such as  [15], [47], [114] or  [115] have been considered. Among these studies, a novel concept of coupling the fields not via a plastic layer but by niobium wires has been tried [115]. Also, pulsed waveforms instead of sine waves, which are dominantly used for capacitive coupling, have been considered [114], [115] (however, it is not entirely clear which waveform has been used in [114]). Likewise, smaller field strengths ranging from 0.35 –  [46], [113], [116] or about  –  [15], [47] have been considered. The required voltages ranged between  [15] and  [46]. Again, the electric field strength values have been determined numerically rather than experimentally.

To our knowledge, only one study uses capacitive electric stimulation of human cells describing an experimental characterisation approach [117]. The authors have proposed an equivalent circuit, replacing the electrode-electrolyte interface with pure capacitors, describing the insulating layer on the electrodes. They measured the impedance of the stimulation device (medium-filled plastic flask with glued electrodes). Moreover, the current density was measured and found to be in agreement with numerically predicted current density values. Recently, a similar approach was established in our lab and could show that exact knowledge of the cell culture medium's conductivity is required to estimate the electric field strength reliably [118].

All studies of capacitive electrical stimulation are summarised in Table 2.

**Table 2:**
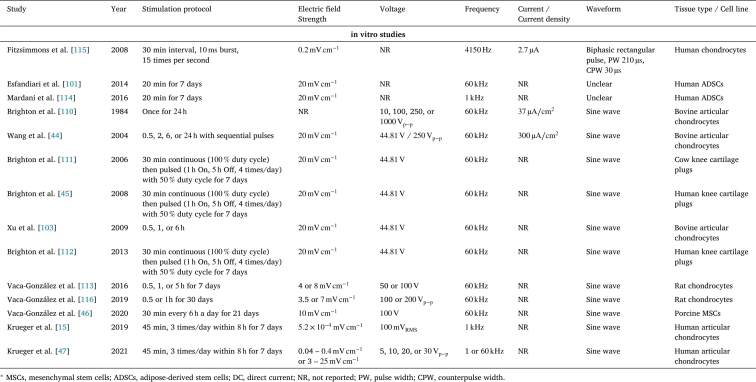
Electrical stimulation studies of articular cartilage using capacitive coupling.

### 3.4 Electrochemical analysis

In the following, all necessary techniques to estimate the electric field strength of the stimulation reliably will be compiled. Firstly, the focus is on direct contact stimulation. Please note that direct contact stimulation is not direct current (DC) stimulation because the term ‘direct contact’ does not provide information about the shape of the stimulation signal but only about the electrode placement. Direct contact stimulation usually leads to electrochemical processes at the electrode surface [120]. In particular, at frequencies in the sub- range, electrical stimulation results in the production of harmful byproducts of electrochemical reactions [121], [122]. Thus, DC signals have not been at the centre of attention in cartilage tissue engineering in recent years due to strong electrochemical reactions. Moreover, the applied current is known to decrease exponentially with time if a constant voltage is applied [71]. Instead, the focus will be on time-harmonic signals (e.g., sine waves or rectangular waves as shown in Fig. 2 and all other waveforms in Table 1, Table 2). For such signals, the analysis of the system response is usually performed in the frequency domain [123]. The impedance expresses the system's response.

The impedance is impacted by multiple parameters. For example, a suitable electrode material can reduce unwanted electrochemical reactions. Unfortunately, it is not possible to reliably predict electrochemical reactions because they usually depend on electrode surface and geometry [122]. Thus, measurements of voltage, current and impedance over a wide frequency range and with different input parameters are required [71], [72], [122]. The response, i.e., the impedance, can be nonlinear with respect to the applied stimulus. This means, in practice, the impedance of the sample and the electrode-sample interface can decrease due to increased electrochemical reactions. Schwan [124] and Richardot et al. [120] have reported detailed theoretical and experimental analyses of the properties of platinum electrodes using electrical stimulation. It is important to determine the limit of linearity, which depends on the frequency and the electrode material [125], [126]. Beyond the voltage amplitude associated with this limit, the impedance is nonlinear. For this reason, it is required to measure the impedance at multiple voltages at a fixed frequency. Roughly speaking, the voltage from which the impedance decreases with increasing voltage is the limit of linearity. Furthermore, the impedance phase can be used as an indicator. As an example of this, the transition from capacitive () to diffusive () characteristics was noticed for a platinum electrode in saline solution the voltage change from  to  at a frequency of  [125].

The measured impedance is described by equivalent circuit models, usually consisting of linear elements only. For example, a constant-phase element is used to describe the electrode-tissue interface. However, nonlinear effects can be introduced into these models by using the Taylor series expansion of the current response [120], [123]. For example, the Butler-Volmer equation has been utilised by Richardot et al. [120] for their nonlinear model, customarily used to represent a reversible electrochemical reaction [123]. Developing such a nonlinear model to describe the electrical stimulation is usually out of scope because all reactants (ions, charged proteins, etc.) in the biological tissue and cell culture medium need to be considered. As there is usually no a priori knowledge of the electrochemical reactions of these reactants, this approach is impractical. Furthermore, nonlinear behaviour indicates electrochemical reactions that might be detrimental, as described above. These reactions require thorough investigation to determine whether the given biological effect of electrical stimulation is related to the products of the electrochemical reactions or to the electrical signal [127], [128], [129].

The electrical stimulation approaches can be linked to electrochemical characterisation methods. Direct current stimulation at a fixed voltage results in the generation of a time-dependent current. In electrochemistry, this monitoring is known as chronoamperometry. Similarly, monitoring the time-dependent voltage at a fixed current is called chronopotentiometry. A related characterisation method is cyclic voltammetry, where the voltage changes between negative and positive values relative to an electrode. The resulting voltage-current graphs are used to identify particular electrochemical reactions at the corresponding voltages [122]. However, the voltage is determined relative to a reference electrode and is not the same as the applied voltage in the stimulation experiment.

Alternating current stimulation using sine or periodic waves resembles measuring the impedance if both voltage and current are recorded. Probing the impedance over an extensive frequency range is called electrochemical impedance spectroscopy (EIS). Only the linear system response should be measured using the EIS technique. To do so, the applied voltage is in the  range to avoid nonlinear electrochemical reactions. In the second step, the impedance measured by EIS is described as an equivalent circuit. Validity tests are available to ensure that such an equivalent circuit can be found [130]. In general, EIS is also applicable for capacitive coupling stimulation. Advantageously, no electrochemical effects at the insulation-sample interface and no nonlinear effects at the electrode are to be expected [131]. Nevertheless, the impedance is expected to be dominated by the insulating layer separating the electrodes and the sample [132]. An introduction to EIS applications with a focus on bioimpedance can be found in the textbooks by Orazem et al. [123] and Grimnes et al. [133]. An excellent introduction to this topic has recently been provided by Wang et al. [134].

In short, a general expected equivalent circuit can be formulated for each stimulation type. This permits the establishment of a corresponding electrochemical measurement approach for each stimulation type [71]. An overview of this relationship and the relevant parameters for the two considered stimulation types is given in Fig. 3.

### 3.5 Towards reliable and controllable electrical stimulation

The comparison of the existing stimulation approaches does not permit to identify a clear formulation of the ‘ideal’ electrical stimulation of cartilage (cf. Table 1, Table 2) despite reports of beneficial effects in vitro and in vivo. A clear objective function must be established in terms of the applied electric parameters. We believe that the key ingredients for future experiments are numerical simulations coupled with physical measurements. Ideally, a digital twin can be established, where a validated numerical model is calibrated by live recordings, providing live information about the stimulation system. Digital twins for in vitro studies have recently been introduced [71], [72] and can straightforwardly be applied to cartilage tissue engineering.

A key ingredient is the impedance spectroscopy of the stimulation system. Ideally, it is conducted with a sample having known properties (e.g., an electrolyte solution) and on the actual sample multiple times (at least before and after the stimulation). Using an equivalent circuit (examples shown in Fig. 3), the sample properties can be distinguished from the electrode-sample interface properties. Simultaneously, the sample impedance is computed using, for example, a freely available finite element method (FEM) library to solve the electroquasistatic field equation, which is suitable for the typical stimulation parameters [136]. Once the measured and the predicted impedance of the sample match with respect to the measurement and model uncertainties (usually, 1% to 5% relative difference [71]), the FEM model can be assumed to be validated. The electrode-sample interface can then be integrated into the numerical model using empirical relations [72], [86], [137], [138]. As the electrochemical interface layer usually has a minimal thickness in the range of a few , it is modelled by a boundary condition. It is numerically too expensive to resolve the interfacial layer spatially because stimulation devices have a characteristic size in the range of  to . Hence, measurements are inevitable to determine each stimulation electrode's electrochemical characteristics and to calibrate the empirical relations.

An open question is how to numerically model the electrode-tissue interface layer. One possibility is to assume a voltage divider, in which the interface impedance is described as a lumped element [86]. In this way it does not affect the field distribution, only the field magnitude and phase. On the other hand, a distributed impedance can be assumed [137]. Ideally, both approaches will give the same result. However, for some electrode configurations, significant differences have been observed and confirmed experimentally [72]. To avoid an influence of the interface, the stimulation should be conducted at frequencies beyond the so-called cutoff frequency. The cutoff frequency marks the frequency from which the sample impedance dominates over the impedance of the electrochemical surface layer. Beyond the cutoff frequency, a validated simulation model is fully predictive. For capacitive coupling, there exists no cutoff frequency, and FEM models usually predict the sample behaviour very well if the geometry and dielectric properties are known [118], [132]. Changes during stimulation can then be detected by live monitoring of the current, impedance and voltage. Examples are changes of the sample temperature [71] or corrosion [72] or mechanical changes [118] of the stimulation electrodes. However, attention must be paid to the magnitude of the observable. For example, capacitive coupling devices usually feature a very large impedance of the insulation layer on the electrodes [132] so that the voltage drop across the cell culture medium is not measurable with conventional voltage probes [139]. Furthermore, temperature-related changes in the impedance of the cell culture medium cannot be detected, given the accuracy of available impedance analysers. These results show that improved stimulation devices that cannot only stimulate but also monitor are required.

In our opinion, the lack of current and voltage recording data (compare Table 1, Table 2) currently marks the biggest problem in the understanding and retrospectively analysing of the electrical stimulation approaches described in the literature. In particular, because doubts have been raised about the reliability of reported stimulation field strengths, retrospective estimates of the field strength are warranted. The current approach of comparing electrical stimulation experiments using only the reported electric field strength (for example, in [36], [38]) does not seem appropriate in this regard. However, retrospective estimates usually require the numerical solution of the electroquasistatic field equation, which requires knowledge of the dielectric properties and the geometry of the stimulation device. For in vitro experiments, usually, only the conductivity but not the permittivity of the cell culture medium needs to be considered [71]. While there are reports on the conductivity of different cell culture media [140], ideally, the conductivity is measured with a conductivity meter. Validated numerical simulations also permit to estimate the conductivity by matching the measured resistance [71], [72]. Attention needs to be paid to the temperature of the cell culture medium, which can strongly influence the measurement result. Unfortunately, the dielectric properties of hydrogels, cartilage, and surrounding tissues are not very well known [141], [142]. In particular, it is unclear how the dielectric properties change over time when a hydrogel or a tissue sample is kept in a cell culture medium. It is known that the water content strongly influences the conductivity of cartilage [143], but consistent measurements over time under cell culture conditions are lacking. To our knowledge, the permittivity of cartilage and hydrogels at relevant frequencies has not been investigated. As a rule of thumb, it can be assumed that the conductivity of cartilage is approximately  to , while cell culture media typically have a conductivity of  to . Other parameters are known from the literature, e.g., that the conductivity of cell culture dishes tends to zero. They can then be modelled as perfect insulators and, in many cases, omitted from the simulation domain. A special case is the dielectric layer in capacitively coupled stimulations. Its material parameters are assumed based on literature values and need to be assessed by uncertainty quantification [132].

**Figure 4:**
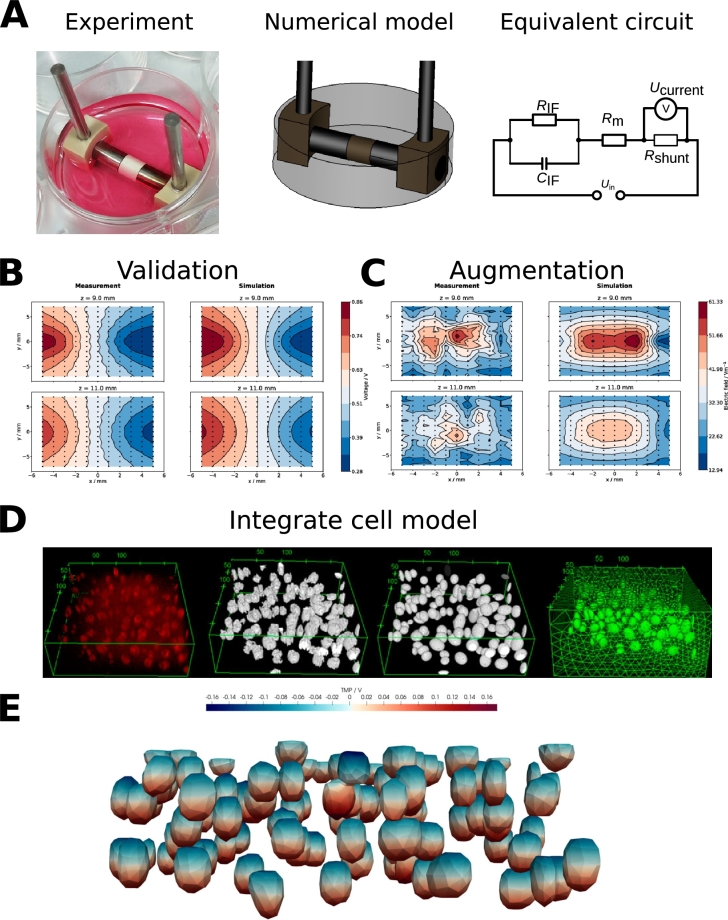
Future vision of cartilage tissue engineering experiments: Experiments are conducted alongside numerical simulations (panel **A**, adapted from [72], it is under CC-BY licence (CC BY 4.0)). A numerical model based on real geometry is built together with an equivalent circuit model. Ideally, the model is also validated by measurements of the local voltage distribution (panel **B**, taken from [72], it is under CC-BY licence (CC BY 4.0)). On the left in panel **B**, the measured voltage is plotted against the simulated voltage on the right. The electric field is computed from the measured and simulated potential distribution (panel **C**, also taken from [72], it is under CC-BY licence (CC BY 4.0)). Note that the field in panel **C** was not computed from the potential in **B**. Instead, an interface impedance was included to augment the model, which led to an asymmetric field distribution in agreement with the experiment. Other modelling choices could not match the experimental observation (for more details see [72]). In the next step, the cell distribution can be inferred from microscopy images (see a workflow in panel **D**, adapted from [145], it is under CC-BY licence (CC BY 4.0)). This permits to estimate the induced transmembrane potential (panel **E**, taken from [145], it is under CC-BY licence (CC BY 4.0)).

The cellular dielectric properties cover a wide range [144], and their influence on the modelling result needs to be carefully tested [109]. In future research, optical methods combined with impedance spectroscopy could close the knowledge gap regarding the dielectric properties of cell-seeded hydrogels and cartilage [142], [145], [146]. Once a digital twin is established, the voltage, electric field and current density, which the cells are locally exposed to, will be known at every time point with a valid data recording. A summary of this vision is presented in Fig. 4.

With this information, more advanced biophysical models can be developed. In the following section, some potential biophysical interactions are introduced. In addition to the biophysical modelling, samples stimulated at the wrong temperature or at reduced field strength due to, for example, corrosion can be excluded from the statistical analysis. Potentially, this can lead to more significant and more explicit evidence of the biological effect of electrical stimulation.

## 4 Biophysical theories of the interaction of electric fields and cells

Various studies have hypothesised that electric fields can stimulate non-excitable cells, e.g. chondrocytes, to differentiate, proliferate or produce chondrogenic proteins. Still, it is unclear which physical processes are causal. Multiple studies have focused on finding correlations between the applied stimulation (current, voltage, electric field) and the cellular response. Selected theories of the interaction between electric fields and cells will be highlighted in the discussion to follow and linked to cartilage stimulation.

### 4.1 Induced transmembrane potential

The cell membrane is a lipid bilayer having a thickness of around  [133], which separates the cell interior (the cytoplasm) from the extracellular medium. Each cell type has a characteristic resting transmembrane potential in equilibrium. The transmembrane potential is the difference between the electric potential on the inner and the outer side of the membrane.

Compared to eukaryotic cells, which have a resting transmembrane potential in the range of  to  [147], chondrocytes have an increased resting potential of  to  [148]. It has been hypothesised that chondrocytes maintain a comparatively ‘positive’ transmembrane potential to bear changes in the osmotic pressure compared to other cells. Importantly, hyperpolarised chondrocytes cannot limit their volume when there is a change of extracellular osmolarity [149], while the depolarisation of chondrocytes results in a reversible volume change.

Voltage-dependent calcium channels are essential in forming and maintaining healthy cartilage [150]. However, their reaction mechanism to external electrical stimuli is unclear [21]. Electrical stimulation can generally produce an induced transmembrane potential, which results in hyperpolarisation (decreased membrane potential) or depolarisation (increased membrane potential). Different voltage-dependent calcium channels exist, which are distinguished as low-voltage and high-voltage-activated channels. Low-voltage-activated pathways open in the  to  range [151]. Larger variations in the transmembrane potential are required for the high-voltage-activated channels. We are not aware of estimated values for the activation of chondrocytes.

Xu et al. have reported that blockers of voltage-dependent calcium channels inhibit entirely the electrical stimulation effects [103]. No estimate of the induced transmembrane potential in adult bovine chondrocytes has been made for their capacitively coupled electrical stimulation device. The voltage-dependent activation of calcium channels has also been investigated for other cell and electrical stimulation types [152], [153].

When even higher membrane potentials are induced (hundreds of ), it comes to electropermeabilisation [147]. The membrane becomes porous due to a strong electric field inside the very thin membrane. The induced membrane potential divided by the membrane thickness can be considered as an estimate of the induced electric field strength.

One can estimate the lower effective range of the induced transmembrane potential by comparing the drift current to the random diffusion current. The applied electric field causes the drift current, while the diffusion current originates from the random thermal motion of the charge carrier ions. At room temperature with initial transmembrane potential values of around , the drift current becomes significant [154]. Then, ions are driven through the cell membrane [155]. For example, the influx of Ca^2+^ ions into bovine endothelial cells has been reported at transmembrane potentials in the  –  range [156]. The influx has also occurred after adding different Ca^2+^ channel blockers, which evidences influx through non-specific membrane pores.

To summarize, the aforementioned observations indicate that very large induced transmembrane potentials greater than  should be avoided in cartilage tissue engineering to not destroy the membrane. Instead, intermediate transmembrane potentials in the range of  to  (to trigger membrane channels) and small transmembrane potentials in the range of a few  (to induce ion migration through the membrane) should be considered for electrical stimulation protocols. Even smaller transmembrane potentials are on the scale of thermal fluctuations and should not be expected to lead to a favourable biological response in most cases.

There exist analytical [157], [158], [159] as well as numerical techniques [160] to estimate the transmembrane potential [109], [145]. An experimental validation of the estimated values has been presented in [160], [161]. Under idealised assumptions, the induced transmembrane potential can be approximated by Schwan's equation and scales linearly with the applied field and the cell radius but is modulated by the cosine of the angle between the field direction and location on the membrane. Thus, distinct ‘hot spots’ exist on the membrane with a maximum value of the transmembrane potential. For the applied time-harmonic signals, the transmembrane potential is also assumed time-harmonic. Hence, it continuously hyper- and depolarises the membrane. The induced transmembrane potential for chondrocytes under capacitive coupling electrical stimulation vanishes if the cell membrane is not in direct contact with the insulation layer or is relatively conductive [109], which shows that numerical models need to be thoroughly scrutinised regarding their uncertainties. In sum, further research on the mechanism of interaction is required.

### 4.2 Electromechanical interaction

The electromechanical interaction of electrical stimulation can occur at the cellular scale, the membrane scale, or even the protein scale.

#### 4.2.1 Cell migration: dielectrophoresis and galvanotaxis

Cell migration due to an external electric field is referred to as electrotaxis or galvanotaxis (direct current fields) and dielectrophoresis (alternating current fields) [162], [163], [164]. Moreover, cells may also rotate due to an externally applied electric field, known as electrorotation. However, this effect is not discussed here as rotating electric fields are needed, which are rare in the context of cartilage tissue engineering.

In electrotaxis, the electric field functions as a compass for the cells [162]. The electric field does not control the cells in a defined direction compared to electrophoresis. Instead, electrotaxis is a biological phenomenon, and depending upon the cell type, both cathodal and anodal migration can be observed irrespective of the field's polarity. For example, electrophoresis theory could have anticipated anodal migration while chondrocytes exhibit cathodal migration [50]. A more recent study has shown that the migration direction depends on the cell passage and that chondrocytes, after the second passage, migrate to the anode [165]. Thus, it is not possible to understand galvanotaxis in the context of electromechanical coupling as it has an active biological component to be considered. As alternating current signals are the typical stimulation signals in cartilage tissue engineering, galvanotaxis can be excluded as a possible reason for the biological effect of electrical stimulation.

Cells can also migrate due to dielectrophoresis upon alternating current electrical stimulation. In contrast to galvanotaxis, dielectrophoresis is explained by classical electrodynamics [163]. The force acting on the cells depends on the electric field strength gradient. Hence, no net force acts on the cell in a spatially constant electric field, so there is no cell migration. Thus, inhomogeneous electric fields are applied in devices for dielectrophoresis. Cells migrate towards higher or lower field strengths, depending on various parameters (e.g., frequency and dielectric properties of the extracellular medium). The effect has been exploited for the cellular organisation of, for example, chondrocytes inside hydrogels [51]. The applied maximum electric field strengths in dielectrophoresis applications are in the  or  with typical frequencies from a few  to  range. Commonly, short signals are used, which are not applied for more than a few minutes [164].

#### 4.2.2 Electro-deformation

Even though homogeneous electric fields can deform the spherical cells, they cannot induce migration of these cells [166], [167]. It has been reported that MSCs embedded in hydrogels stimulated by the capacitively coupled electric field (, ) change into more spherical shape in contrast to the control sample [46]. Similarly, directly coupled electric field ( at ) changes the human MSCs into circular shape [168]. Surprisingly, capacitive electric stimulation has enhanced chondrogenic differentiation [46] compared to direct contact electric stimulation, favouring osteogenic differentiation [168]. A further aspect of the electro-mechanical interaction is the piezoelectric-like behaviour of cartilage. Frank et al. have shown that an externally applied current density leads to the deformation of cartilage samples [74], [75], which can further impact the cartilage cells.

To explain the electro-deformation phenomenon of cells, giant vesicles (cell-like constructs usually having no nucleus) have been studied [169]. The morphological changes in alternating current fields are based upon the conductivity ratio (i.e., the conductivity ratio inside and outside the cell) [170]. Four shape transitions exist at different frequencies and conductivity ratios: prolate-to-spherical, oblate-to-spherical, oblate-to-prolate, and prolate-to-oblate. They could be described by models based on electrohydrodynamics [171] or based on energy considerations [172], [173]. An important future direction will be to observe the morphological response of cartilage cells under electrical stimulation and to build reliable numerical models. As improved chondrogenesis has been linked to a spherical cell type [174], numerical simulations aiming to optimise cell shape could provide a means to develop improved stimulation settings.

#### 4.2.3 Interaction with membrane constituents

The effect of oscillating electric fields could be related to electroconformational coupling, which has been explored since the late 1980s [175]. However, the effect has not been visually observed but was inferred from a nonlinear response measured by dielectric spectroscopy applying an electric field on yeast cells with, for example,  at  [176] or  at  [177]. The same response in yeast cells has been observed by measuring the magnetic field produced by the time-dependent current [178], [179]. However, it is worth mentioning that there exist conflicting experimental results, and the non-linear response has not always been observed [180], [181].

The biological effect of the electroconformational coupling has been linked to H^+^-ATPase membrane pumps [176], also affected by the membrane potential [182]. Sodium potassium pumps (Na^+^/K^+^-ATPase) are described in chondrocytes [183] and are essential for cartilage tissue engineering [184]. However, their relation to the external electric field is not known yet.

Another open research topic is the motility of membrane constituents. For example, lipid rafts have been shown to cluster upon electrical stimulation in fibroblasts [185]. At extremely low frequencies (), an increased induced transmembrane potential is generated due to the rearrangement of receptors [186]. The primary cilium, a membrane protrusion in chondrocytes, could also respond to electrical stimulation [187]. Its role in mechanotransduction and the interaction with external electric fields is the subject of ongoing research.

Moreover, chondrocytes also express Piezo channels [188], [189] or TRPV4 [190] mechanosensitive membrane channels. The mechanism of interaction of the membrane and its constituents with electrical stimulation is an open research question. Yet, there is no clear understanding of the membrane processes that could be beneficial for cartilage tissue engineering under electrical stimulation. It is hypothesized that the electrical stimulation can trigger stretch-activated calcium channels as well [36], leading to an increased Ca^2+^ influx. Brighton et al. [45], [103] first set out to find what was actually happening at the intracellular level. They reported that the effect of electrical stimulation involves extracellular Ca^2+^ influx through voltage-gated calcium channels. The increased intracellular calcium concentration was found to lead to the activation of calmodulin and then calcineurin, followed by the activation of transcription factors such as SOX9 and nuclear factor of activated T-cells (NF-AT) [191]. Finally, it leads to the expression of genes that are responsible for growth factors such as transforming growth factor-beta (TGF-*β*), collagen type II (COL2), and bone morphogenetic proteins (BMPs) [192], [193], among others. These growth factors are responsible for cartilage repair and regeneration. [194].

## 5 Summary and outlook

We discussed electrical stimulation as a biophysical intervention for the repair and regeneration of cartilage. Despite decades of research, it appears that no reliable electrical stimulation protocol for application in cartilage tissue engineering has been established. Two principal parameter combinations may be considered: direct contact versus capacitive coupling stimulation and alternating current versus direct current stimulation signals. Nevertheless, the search for the most effective stimulation parameters remains an open question. The selection of alternating current signals over direct current signals is supported by evidence of increased biocompatibility. Furthermore, the selection of stimulation parameters through trial and error has not resulted in the establishment of well-defined stimulation protocols suitable for clinical translation. Electrical stimulation can influence chondrocytes at various levels, including mechanical impact through induced migration or deformation. Additionally, intracellular processes may take place that regulate the individual cell fate, such as proliferation and differentiation. Despite extensive research, the precise mechanism of interaction of electrical stimulation in cartilage tissue engineering remains elusive.

We believe that improved reporting standards focusing on technical replicability combined with continuous monitoring of stimulation parameters will eventually enable a more effective determination of optimal stimulation protocols. In this regard, numerical simulations will play a pivotal role in understanding the interaction of cartilage and its chondrocytes with an externally applied electric field. The bidirectional exchange of information between numerical models and experimental measurements, also called digital twin, will accompany these efforts. We highlighted biophysical theories of the interaction between electric fields and cells that can be used to design hypothesis-driven stimulation experiments. Thanks to ever-improving experimental techniques, closer integration of observations on multiple scales will enable tailored cartilage tissue engineering protocols.

## CRediT authorship contribution statement

**Julius Zimmermann:** Writing – review & editing, Writing – original draft, Visualization, Validation, Software, Resources, Methodology, Investigation, Formal analysis, Data curation, Conceptualization. **Abdul Razzaq Farooqi:** Writing – review & editing, Writing – original draft, Visualization, Validation, Software, Methodology, Investigation, Formal analysis, Data curation, Conceptualization. **Ursula van Rienen:** Writing – review & editing, Supervision, Project administration, Funding acquisition, Conceptualization.

## Declaration of Competing Interest

The authors declare that they have no known competing financial interests or personal relationships that could have appeared to influence the work reported in this paper.
